# Risk factors of pelvic organ prolapse at Asella Teaching and Referral Hospital: Unmatched case control study

**DOI:** 10.3389/fgwh.2022.833823

**Published:** 2022-09-13

**Authors:** Mohammed Suleiman Obsa, Tahir A. Worji, Nemo A. Kedir, Negeso G. Kute

**Affiliations:** ^1^Department of Anesthesia, Wolaita Sodo University, Sodo, Ethiopia; ^2^School of Medicine, Arsi University, Asella, Ethiopia; ^3^Department of Anesthesia, Hawassa University, Hawassa, Ethiopia

**Keywords:** pelvic organ prolapse, pelvic floor dysfunction, maternal health, risk factors, Asella

## Abstract

**Background:**

Prolapse is one of the sub-types of pelvic floor dysfunction (PFD) which occurs due to abnormal fall of the pelvic organs from their normal anatomic positions. Although the cause of prolapse is multifactorial, it primarily occurs due to pregnancy and vaginal delivery. Hence, the present study aimed to identify risk factors of prolapse among women who undergo gynecological surgery.

**Materials and methods:**

Facility-based-unmatched case–control design was employed. Cases were all gynecological women who were diagnosed with pelvic organ prolapse (POP) at Asella teaching referral hospital (ATRH) while controls were all charts of gynecological women who were diagnosed with other gynecological problems rather than POP at ATRH. For each case, two controls were selected using a simple random sampling technique. The data were entered into Epidata version 4.3.1 and finally exported to SPSS version 25 for further analysis. Then variables that had an association in the bivariate model (*p* < 0.25) were entered and analyzed by a multivariable conditional logistic regression model to identify the independent effect of different factors. Statistical significance was declared at *p* < 0.05.

**Results:**

A total of 147 cases and 293 controls were included in this study. Women who had a history of chronic cough, previous pelvic floor surgery, constipation, and vaginal tear during delivery, history of pelvic trauma, age of the women, rural resident, and maternal gravidity were strongly associated with prolapse at *p*-value of < 0.05. Multigravida [adjusted odds ratio (*AOR*) 2.987 (95% *CI* 1.237–6.853), *p* = 0.014], age >50 years [*AOR*: 2.496 (95% *CI* 1.372–4.539), *p* = 0.003], women with a history of pelvic floor surgery [*AOR*: 0.3.666 (95% *CI* 1.328–10.124), *p* = 0.012], women who had diabetes mellitus [*AOR*: 4.676 (95% *CI* 0.908–24.075), *p* = 0.065], and resided in rural areas [*AOR* = 1.878; (95% *CI*: 0.984–3.585), *I*^2^ = 47.5%, *p* = 0.056] were the independent predictors were of prolapse.

**Conclusions:**

In this study, women with diabetes mellitus, previous pelvic floor surgery, rural residents, being multigravida, and age >40 were independent predictors of prolapse. Therefore, delivering health education by focusing on the identified risk factors was strongly recommended.

## Introduction

Prolapse occurs when abnormal descent of the pelvic organs occurs from their normal anatomic positions. It is primarily a common gynecological condition that is considered as a medical and social problem, deeply rooted with poor health services and socio-cultural beliefs affecting women in childbearing age and post-menopausal age (1, 2). Patients generally present with several complaints, such as bladder, bowel, and pelvic symptoms; however, with the exception of vaginal bulging, none is specific to prolapse. Women with symptoms suggestive of prolapse should undergo pelvic examination and medical history checkups. However, many patients with pelvic organ prolapse are asymptomatic and do not need treatment (3). In a general population, only 12% of women aged between 45 and 85 years women are symptomatic though over two-thirds of these women have anatomical evidence of pelvic organ prolapse (POP) (4, 5). However, women with symptomatic pelvic floor dysfunction PFD suffer from physical and emotional distress which has a great negative impact on women's social, physical, and psychological wellbeing (6, 7).

Therapeutic options for POP include surgery and conservative treatments. Although surgical management of POP is currently adopted, non-surgical treatments such as pessaries, pelvic floor muscle training, or both can be useful in symptomatic improvement (8, 9) as well as weight loss in case of obesity. Nevertheless, most of these treatments are not helpful for women with severe prolapse; therefore, surgical therapy is more appropriate in these cases. The surgical management, depending on the type of POP, includes apical suspension (sacral colpopexy and sacrospinous ligament fixation), anterior and posterior (colporrhaphy, perineorrhaphy, and obliterative procedures) vaginal prolapse repair (8).

Surgical repair is the first choice of treatment in case of severe POP (stage III–IV, according to the International Continence Society POP-Q classification (10). Surgery usually includes hysterectomy, performed through different approaches (vaginal, laparoscopic/robotic, and abdominal) (11). The two most accepted surgical techniques for primary VPP are laparoscopic sacrocolpopexy (LSC) and sacrospinous fixation (SF) (12). The second recurrence of vaginal vault prolapse (VVP) is defined as prolapse of the vaginal vault or upper vagina after two previous reconstructive surgeries. The recurrence of VVP occurs when the top of the vagina descends below a point that is 2 cm less than the total vaginal length above the plane of the hymen (13).

Although the exact prevalence of pelvic organ prolapse is unknown, the lifetime risk of requiring at least one operation to correct prolapse has been roughly estimated at 11% (14). Prolapse procedures are known to have a high reoperation rate, with a lifetime risk for surgery of 10–20% (15). Although several approaches are available for the management of POP, the best strategy in case of recurrence after vaginal vault prolapse still remains debated (16, 17). However, it is assumed that the success rate of POP surgery would increase by combining surgery with PFMT (18). Recent systematic reviews have concluded that PFMT reduces POP symptoms and severity stage (17). PFMT has been shown to increase pelvic floor muscle (PFM) strength and endurance, reduce the levator hiatus area, lift the bladder and rectal ampulla, increase PFM volume, and reduce PFM length (18).

The cause of prolapse is primarily related with pregnancy and vaginal delivery, which lead to direct pelvic floor muscle and connective tissue injury. These defects may be due to stretching and tearing of the endopelvic fascia, levator muscles, and perineal body during childbirth (19). The combinations of anatomical, physiological, genetic, lifestyle, and reproductive factors that interact throughout a woman's lifespan also contribute to PFD. Hysterectomy, pelvic surgery, and conditions associated with sustained episodes of increased intra-abdominal pressure, such as obesity, chronic cough, constipation, and repeated heavy lifting, also contribute to prolapse (20).

The prolapse affects severely affects women's quality of life in several ways. Women with POP can feel different prolapse symptoms like “something coming down” and other urinary, bowel, and sexual symptoms. It has socioeconomic and health consequences, affecting overall health and sexual function (21). These women frequently report disorders of sexual desire, arousal, orgasm, and pain and these problems can decrease the quality of life and affect the relationship between partners (22). The disease impairs healthcare seeking behavior of women due to a series of socio-cultural myths, lack of familial support, treatment cost, women's reluctance and wrong perception of the prolapse as a malignancy (23).

A previous study conducted in northern Ethiopia showed that sphincter damage, family history of POP, being uneducated, having ≥ 4 vaginal deliveries, carrying heavy objects, maternal gravidity, and BMI < 18.5 kg/m^2^ as determinants of POP (24). However, this study identified additional factors like chronic cough, previous pelvic floor surgery, constipation, vaginal tear during delivery, history of trauma, age of the women, rural resident as associated and, women with diabetes mellitus, previous pelvic floor surgery, rural resident, being multigravida and age >40 years as the independent predictors of POP. Besides, there may be different in sociodemographic, socioeconomic, and lifestyles differences between south-eastern Ethiopia and northern Ethiopia. Hence, this study aimed to identify risk factors of pelvic organ prolapse among gynecological patients who underwent surgery at Asella Teaching and Referral Hospital in order to segment interventional on identified risk factors.

## Materials and methods

This retrospective unmatched case–control study was conducted using a simple random sampling technique from February to March 2021 at Asella Teaching Referral Hospital (ATRH), South East Ethiopia. Age at the first delivery was used to calculate the final sample size as it gave the maximum sample size, 440 [147 cases and 293 controls] with the following assumptions (24): 91.9% proportion of exposed control, 64.9% proportion of case (24), 95% confidence interval (CI), 80% power, 6.1 odds ratio, 2:1 controls to cases ratio. The case definition of this study was charts of women who reported to have at least one of the pelvic floor disorders (*utero*-vaginal prolapse, rectocele, cystocele, vault prolapse, and delivered myoma) with stages two and above (23). On the other hand, all charts of women who were diagnosed gynecological problems other than pelvic floor disorders were controls. In this study charts of women with both pelvic floor disorder and other gynecological problems, charts of women with stage one pelvic floor disorder and charts with at least three incomplete identified risk factors were excluded. Before selecting cases and controls, all 2 years charts of women with all gynecological problems were identified. After that, a simple random sampling technique was used to separately select both cases and controls from its respective group. Four nurse degree holders' and two masters of Science degree holders were recruited as data collectors and supervisors, respectively. The training was given for the data collectors and supervisors for 2 days. The aim of the training was to make understanding on the objective of the study, data collection tool, data quality assurance, and data collection procedures. Data were entered into Epidata version 4.3.1 and then exported to Stata v14.0 (Statacorp, College Station, Texas, USA) software for analyses. Then variables that had an association in the bivariate model (*p* < 0.25) were transported and analyzed by a multivariable logistic regression model to identify the independent effect of different factors. A stepwise approach will be performed to select variables for inclusion in modeling. Statistical significance will be declared at *p* < 0.05. To check co-linearity between risk factors, tolerance and variance inflation factor (VIF) were used (25). Adjusted odds ratio (*AOR*) with a 95% *CI* was used to measure the strength of association. Calibration of the model was determined by a non-significant Hosmer–Lemeshow goodness of fit test (26).

## Result

### Sociodemographic characteristics of the patient

A total of 440 women were included in this case–control study to determine risk factors of pelvic organ prolapse. The majority of the patients with pelvic organ prolapse were found in the age >50 years and the lowest percentage of women were found between the age group of 18–24 years. The mean age of respondents was 35.3386 ± *SD* (10.77292), (minimum 18 and maximum 65). In addition, the majority of women were from rural areas 343 (77.95%). The regarding the residence of women, about 94.55% of all patients were from rural areas while the remaining 22% were from urban. Majorities of the patients 94.5% of all women were married (Table 1).

**Table 1:** Sociodemographic of patients with pelvic organ prolapse at Asella Teaching Referral Hospital (ATRH), 2021.

**Variable**	**Category**	**Frequency**	**Percentage**
Marital status	Single	23	5.22
	Married	416	94.55
Residence	Urban	97	22.05
	Rural	343	77.95
Age of respondent	18–24	47	10.70
	25–29	67	15.22
	30–34	59	13.41
	35–39	99	22.5
	40–44	28	6.36
	45–49	65	14.77
	>50	75	17.05

### Bivariate analysis on sociodemographic, medical, and other risk factors of POP

Among all sociodemographic and other determinants, intra-abdominal mass and marital status were not associated with POP on bivariable analysis at *p* value of <0.25; hence excluded from the multivariable analysis. On the other hand, sociodemographic and other factors which were associated on bivariable analysis were history of chronic cough, constipation, history of walking long distance, history of carrying heavy wood, history of trauma, age of the women, and resident of women (Table 2).

**Table 2:** Bivariable analyses of sociodemographic, medical, and other risk factors of POP at ATRH, 2021.

**Variables**		**Case**	**Control**	**Sig**.	**Exp(B)**	**95% C.I. for EXP(B)**	
						**Lower**	**Upper**
Age category	>40	54	206	0.000	2.756	1.661	4.573
	<40	93	87	0.564	0.875		
Residence	Rural	23	74	0.023	1.822	1.086	3.055
	Urban	124	219	0.000	1.766		
Marital status	Yes	5	19	0.287	1.969	0.720	5.384
	No	142	274	0.000	1.930		
Intra-abdominal mass	Yes	81	140	0.261	0.775	0.520	1.156
	No	66	151	0.000	2.258		
Diabetes mellitus	Yes	10	10	0.112	0.482	0.196	1.186
	No	136	282	0.000	2.074		
Chronic cough	Yes	54	41	0.000	0.280	0.175	0.449
	No	93	252	0.000	2.710		
Walking long distance	Yes	75	92	0.000	0.439	0.293	0.660
	No	72	201	0.000	2.792		
Carrying heavy wood	Yes	66	69	0.000	0.378	0.248	0.577
	No	81	224	0.000	2.765		
History of pelvic trauma	Yes	18	8	0.000	0.198	0.084	0.467
	No	127	285	0.000	2.244		
Chronic constipation	Yes	38	20	0.000	0.210	0.117	0.377
	No	109	273	0.000	2.505		

### Obstetrics and surgery-related risk factors

All obstetrics- and gynecological-related determinants of POP were associated on bivariable analysis at *p* value of <0.25; hence transported to multivariable analysis. These factors include previous pelvic floor surgery, vaginal tear during delivery, operative vaginal delivery, birth weight, history of prolonged labor, history, home delivery, maternal parity, and gravidity (Table 3).

**Table 3:** Obstetric and surgical risk factors of POP at ATRH, 2021.

**Variables**		**Case**	**Control**	**Sig**.	**Exp(B)**	**95% C.I. for EXP(B)**	
						**Lower**	**Upper**
Pelvic floor surgery	Yes	16	18	0.077	0.530	0.262	1.072
	No	129	274	0.000	2.124		
Operative delivery	Yes	15	9	0.003	0.275	0.117	0.644
	No	130	284	0.000	2.185		
Vaginal tear	Yes	33	14	0.000	0.170	0.088	0.330
	No	112	279	0.000	2.491		
Prolonged labor	Yes	51	35	0.000	0.255	0.156	0.417
	No	96	258	0.000	2.687		
Home delivery	Yes	53	45	0.000	0.322	0.203	0.511
	No	94	248	0.000	2.638		
Macrocosmic baby	Yes	20	7	0.000	0.155	0.064	0.377
	No	127	286	0.000	2.252		
Multipara	Multipara	30	76	0.202	1.366	0.846	2.204
	Nulliparous	117	217	0.000	1.855		
Gravidity	Primigravida	9	52	0.000			
	Multigravida	104	207	0.000	5.778	2.463	13.551
	Grandmultipara	34	34	0.011	1.990	1.171	3.383

### Risk factors of pelvic organ prolapse

In this study, women who had a history of chronic cough, previous pelvic floor surgery, constipation, vaginal tear during delivery, history of trauma, age of the women, rural resident, and maternal gravidity were strongly associated with pelvic organ prolapse at *p* value of <0.05. The result of multivariable analysis also showed women with diabetes mellitus, previous pelvic floor surgery, rural residents, being multigravida, and age >40 years independent predictors of factors of pelvic organ prolapse. Women who had diabetes mellitus had about five times more likely to have pelvic organ prolapse than patients who had no diabetes mellitus [(*AOR*: 4.676 (95% *CI* 0.908–24.075), *p* = 0.065].

The odds of having pelvic organ prolapse were two times more prevalent among women who resided in rural areas than those who were living in urban areas [(*AOR* = 1.878; 95% *CI*: 0.984–3.585), *I*^2^ = 47.5%, *p* = 0.056].

Women who had a history of walking long distance were 89.3% more likely to develop POP than those who were not [*AOR*: 0.893 (95% *CI* 0.437–1.825), *p* = 757]. Additionally, women who had a history of carrying heavy wood were about 105.8 more likely to develop POP than who were not [*AOR*: 1.058 (95% *CI* 0.463–2.422), *p* = 0.893].

Being multigravida women had about three times more likely to develop POP when compared to single gestation [*AOR* 2.91 (95% *CI* (1.237–6.853)], *p* = 0.014). The odds of having pelvic organ prolapse were about 2.5 and 3.7 times among women aged >50 years and women with a history of pelvic floor surgery [*AOR*: 2.496 (95% *CI* 1.372–4.539), *p* = 0.003] and [*AOR*: 0.3.666 (95% *CI* 1.328–10.124), *p* = 0.012], respectively (Table 4).

**Table 4:** Risk of pelvic organ prolapse at ATRH, 2021.

**Variables in the Equation**	**B**	**S.E**.	**Wald**	**D.f**	**Sig**.	**Exp(B)**	**95% C.I. for EXP(B)**	
							**Lower**	**Upper**
Diabetes mellitus	1.542	0.836	3.404	1	0.065	4.676	0.908	24.075
History of chronic cough	−0.884	0.360	6.041	1	0.014	0.413	0.204	0.836
Walking long distance	−0.113	0.364	0.096	1	0.757	0.893	0.437	1.825
History of carrying heavy wood	0.057	0.422	0.018	1	0.893	1.058	0.463	2.422
Pelvic floor surgery	1.299	0.518	6.285	1	0.012	3.666	1.328	10.124
Operative vaginal delivery	−1.181	0.618	3.649	1	0.05	0.307	0.091	1.031
Vaginal tear during delivery	−1.435	0.508	7.979	1	0.005	0.238	0.088	0.645
History of prolonged labor	0.075	0.413	0.033	1	0.856	1.078	0.480	2.422
History of home delivery	−0.552	0.291	3.586	1	0.05	0.576	0.325	1.020
History of pelvic trauma	−1.303	0.659	3.909	1	0.048	0.272	0.075	0.989
Macrocosmic baby	−0.732	0.681	1.153	1	0.283	0.481	0.127	1.829
Chronic constipation	−0.912	0.411	4.939	1	0.026	0.402	0.180	0.898
Age >50 years	0.915	0.305	8.986	1	0.003	2.496	1.372	4.539
Multigravida	1.069	0.437	5.987	1	0.014	2.911	1.237	6.853
Rural resident	0.630	0.330	3.647	1	0.056	1.878	0.984	3.585

## Discussion

Pelvic organ prolapse is downward descent of female pelvic organs, such as the bladder, uterus or post-hysterectomy vaginal cuff, and the small or large bowel, resulting in protrusion of the vagina, uterus, or both. The most valid symptom of POP is the sensation of a bulge in the vagina (27). It is a major female health problem that causes considerable physical and emotional distress, bothers quality of life and influence a large financial burden (15). The effect of disorder is not only limited to the physical health, sexual lives, ability to work, and earn a livelihood of the individual women, but also it affects their families, caregivers, and society at large (22, 24). Hence, women want to preserve their physique and capacity for sexual function well beyond menopause (1).

This study showed that previous pelvic floor surgery was independent predictors of POP which is in line with other studies (28, 29). Another population-based study has shown that at least 30% of women treated surgically for pelvic organ prolapse, urinary incontinence, or both will require subsequent surgery for a recurrence of these conditions (30, 31). It was also founded by another study that the first vaginal delivery and forceps delivery are risk factors of POP (32). In contrast to the current study, operative vaginal delivery and vaginal tear during birth were not associated with POP (4, 33). On the other hand, elective cesarean delivery was protective when compared with spontaneous or operative vaginal delivery (19).

The present study found that multiparty was one of the independent predictors of POP which agrees with other similar studies (34–36). It was also revealed by other studies that pregnancy and childbirth are considered as risk factors for POP (32, 33). This might be due to the fact that repeated pregnancy and birth damages sphincter muscles and ligaments, which sometimes never fully regain its strength and elasticity. However, another study found that operative vaginal delivery other than forceps delivery, age at last delivery, and gravidity were not significantly associated with POP (35).

Like other several studies, we found that the risk of POP increases with age (33, 37–43). Similarly, another study conducted in Jimma, southwest Ethiopia revealed that women aged ≥ 40 years were about three times more likely to have had POP compared to its counterpart (38). The findings of the review article showed that odds of having pelvic organ prolapse were about seven times more likely among women having more than 40 years old than in the younger population (23). The increase in prevalence of POP as age increases might be due the weakening sphincter muscles and surrounding tissues as the age increases (44, 45).

According to the findings of this study, rural resident was independent predictors of POP which agrees with another study (38). A systematic review and meta-analysis done on the burden of pelvic organ prolapse in Ethiopia showed that the odds of having pelvic organ prolapse were 3.29 times more prevalent among women who resided in rural areas than those who were living in urban areas (23). This might be due to the fact that rural women had been assisting in farmland, marketing, wood and water fetching, child rearing and carrying the baby on the back even during pregnancy which has detrimental effects for the loss of genitourinary supporting structures.

The finding of the current study also revealed that chronic cough and chronic constipation were strongly associated with POP which agrees with another study (46). In contrast, other studies found that chronic cough and chronic constipation were associated with prolapse (33, 41, 47). These might be due to the fact that conditions such as chronic cough, constipation, and obesity may predispose some women to disruption, stretching, or dysfunction of the levator anti complex, connective-tissue attachments of the vagina, or both, resulting in prolapse (46).

In this study, diabetes mellitus was identified as among independent predictors of POP. Similarly, another study found that diabetes mellitus was significantly associated with primary POP (4, 41). Other studies also revealed that obesity (BMI ≥ 25 kg/m^2^) could increase the risk of POP (48, 49). The findings of systematic review and meta-analysis revealed a contrary finding that being underweight (BMI, 18.5 kg/m^2^) increases the risk of POP by a threefold (48, 50). However, we did not collect BMI of the patient.

### Limitations of this study

The main limitations of the present study included failure to assess some important variables like age at first delivery, age at last delivery, and BMI of women as our data were secondary source. However, we tried to assess all other documented factors and important characteristics.

## Conclusions

In conclusion, women who had a history of chronic cough, previous pelvic floor surgery, constipation, vaginal tear during delivery, history of trauma, age of the women, rural residents, and being gravida women were strongly associated with prolapse. And the independent predictors of POP were women with diabetes mellitus, previous pelvic floor surgery, rural residents, being multigravida, and age >40 years. Therefore, delivering health education by focusing on the identified risk factors was strongly recommended. Further, multicenter cohort studies with a higher sample size should be conducted to further investigate the risk factors responsible for occurrences of POP.

## Data availability statement

The original contributions presented in the study are included in the article/supplementary material, further inquiries can be directed to the corresponding author.

## Author contributions

MO and TW were involved in the conception, study design, execution, acquisition of data, analysis and interpretation of data, took part in drafting the article, and revising it critically for important intellectual content. NGK and NAK were involved in study design, execution, acquisition of data, analysis, interpretation, drafted, and final manuscript writing. All authors reviewed and agreed on all versions of the manuscript before submission, agreed to submit to the current journal, gave final approval of the version to be published, and agree to be accountable for all aspects of the work.

## Conflict of interest

The authors declare that the research was conducted in the absence of any commercial or financial relationships that could be construed as a potential conflict of interest.

## Publisher's note

All claims expressed in this article are solely those of the authors and do not necessarily represent those of their affiliated organizations, or those of the publisher, the editors and the reviewers. Any product that may be evaluated in this article, or claim that may be made by its manufacturer, is not guaranteed or endorsed by the publisher.

## Abbreviations

ATRH: Asella Teaching Referral Hospital
BMI: body mass index
UVP: *utero* vaginal prolapse.
